# Cavernous internal carotid artery aneurysm presenting with ipsilateral oculomotor nerve palsy: A case report

**DOI:** 10.1016/j.radcr.2021.03.008

**Published:** 2021-04-06

**Authors:** Nizar Almaghrabi, Yousef Fatani, Abeer Saab

**Affiliations:** a Radiology resident, King Abdulaziz Hospital, Makkah, Saudi Arabia; b Neuroradiology Consultant, King Abdulaziz Hospital, Makkah, Saudi Arabia

**Keywords:** Neuroradiology, Oculomotor, Palsy, Cavernous, Carotid, Aneurysm

## Abstract

The oculomotor nerve palsy is a rare neurological deficit, it is associated with numerous underlying pathologies. Including stroke, neoplasms, trauma, post-surgical inflammation, and microvascular damage from chronic disease. It can cause a set of neurological deficits, including diplopia from oculomotor nerve involvement, decreased visual acuity from optic neuropathy, facial hypoesthesia from involvement of the trigeminal nerve, and less frequently facial pain.

We present a case of 52 years old female patient who presented with a history of lateral divination of the left eye associated with ipsilateral drooping of upper eyelid, visual disturbance, and ‏pupil dysfunction.

MRI and MRA were performed and in conventional sequences plus 3D FIESTA sequence and it shows a signal void structure, compressing the left oculomotor nerve after passing through left chiasmatic cistern and upon entrance to cavernous sinus. Reformatted images demonstrate that this structure arising from distal left internal carotid artery at lateral part of cavernous sinus represents a saccular aneurysm in the cavernous part of the internal carotid.

Aneurysms can cause direct compression of the third cranial nerve either by the enlargement of an unruptured aneurysm or by rupture of the aneurysmal sac resulting in third cranial nerve palsy.

## Introduction

Aneurysms of the cavernous portion of carotid artery are uncommon, announcing prevalence between 0.3% and 1.4% of all intracranial aneurysms [1]. These aneurysms are more widespread among females and can be due to idiopathic, infections or trauma.

Internal carotid artery aneurysms that arising from the cavernous part may produce a mass effect and consequent a set of neurological deficits, including diplopia from oculomotor nerve involvement, decreased visual acuity from optic neuropathy, facial hypoesthesia from involvement of the trigeminal nerve, and less frequently facial pain [2]. complications of cavernous carotid aneurysms are infrequent, although it has been reported, which include rupture into the cavernous sinus, causing the formation of a carotid-cavernous fistula, or subarachnoid hemorrhage. Other than spontaneous rupture, complications including progressive compression of cranial nerves in the cavernous sinus.

Oculomotor nerve palsy has been classically divided into pupil sparing and non-pupil sparing. Common causes for pupil-sparing pathologies including diabetic neuropathy, myasthenia gravis, atherosclerosis, chronic progressive ophthalmoplegias [3]. On the other hand, non-pupil sparing oculomotor palsy pathologies include tumor (chordomas, clival meningiomas) and it's the most common cause, followed by vascular lesions (posterior communicating aneurysms and then distal basilar artery aneurysms) [4], Even rarer presentations are uncal herniation and, more rarely (5%) [3], cavernous sinus lesions (vascular pathologies and tumors).

Aneurysms can cause direct compression of the third cranial nerve resulting in third cranial nerve palsy, caused either by the enlargement of an unruptured aneurysm or by rupture of the aneurysmal sac [5]. Some studies mention that the pulsatility and compressive mass effect have been thought of to be the most important causes of third nerve palsy secondary to an unruptured aneurysm [6,7]. However, some articles have elucidated that the size of the aneurysm is not related to the third nerve palsy and that even a relatively small aneurysm may cause third nerve palsy [8], [9], [10].

## Case presentation

A 52 years old female patient presented with a history of lateral divination of the left eye associated with ipsilateral drooping of upper eyelid, visual disturbance, and ‏pupil dysfunction.

The physician suspected a stroke with oculomotor nerve palsy at the time of presentation.

**Fig. 1:**
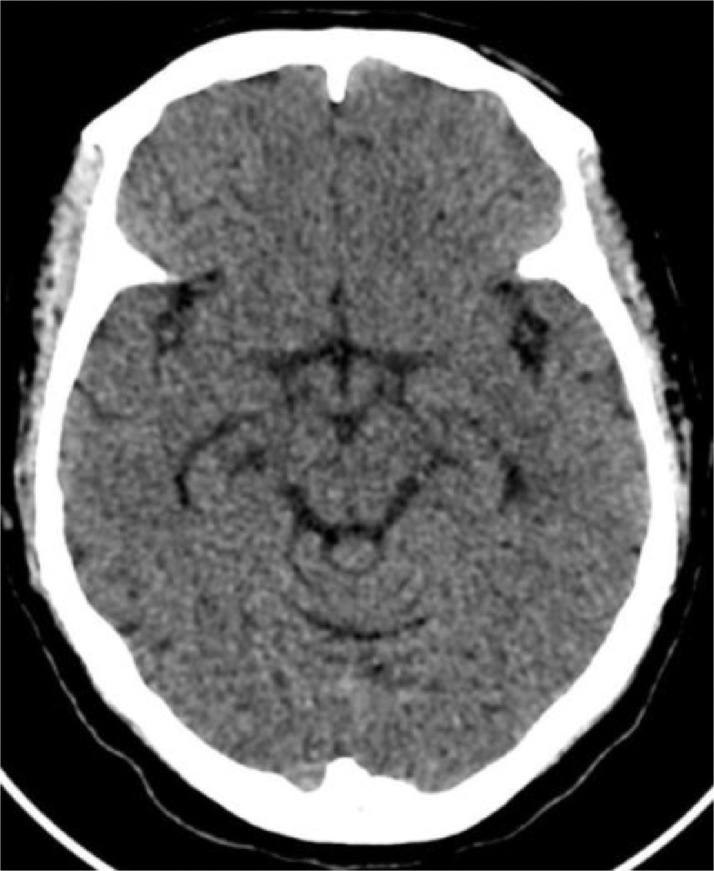
Axial CT image of the brain without contrast was unremarkable.

**Fig. 2:**
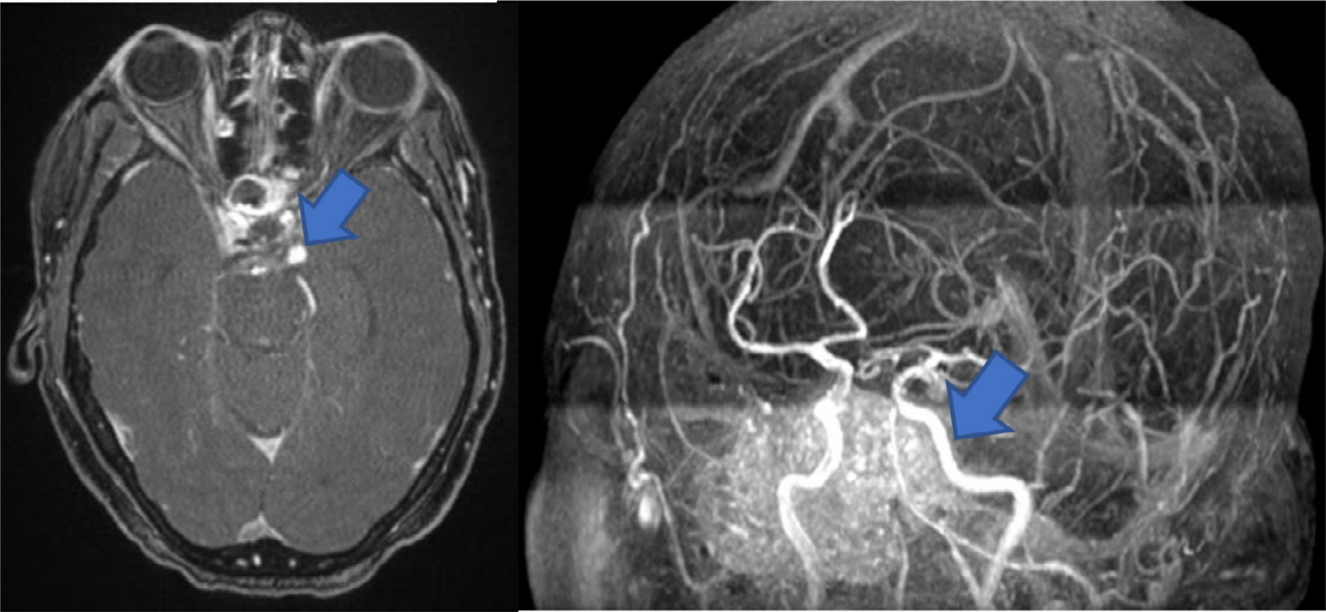
Axial magnetic resonance angiogram with contrast and 3D TOF image demonstrating saccular aneurysm (blue arrow) in the distal left internal carotid artery at lateral part of cavernous sinus (color version of figure is available online.)

**Fig. 3:**
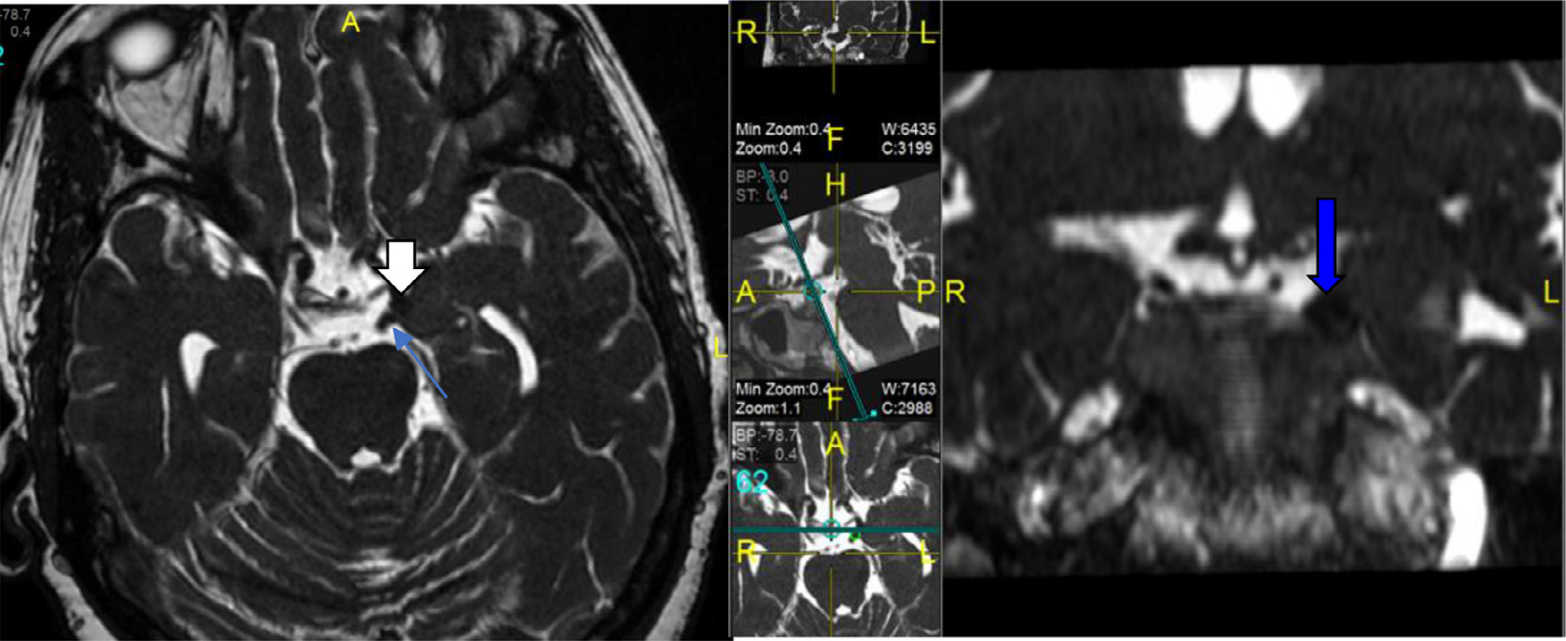
Axial and coronal FIESTA sequence magnetic resonance image demonstrating compression of left oculomotor nerve (blue arrow) by saccular aneurysm (white arrow) (color version of figure is available online.)

Brain MRI and MRA were performed few hours after unremarkable CT brain for stroke (Fig. 1) and it obtained in conventional sequences plus 3D FIESTA sequence with reformatting which revealed no evidence of acute stroke or restriction of diffusion however, (Fig. 3) there is a signal void structure, compressing the left oculomotor nerve after passing through left chiasmatic cistern and upon entrance to cavernous sinus. Reformatted images demonstrate that this structure arising from distal left internal carotid artery at lateral part of cavernous sinus likely represents a saccular aneurysm which is confirmed by Magnetic resonance angiography (MRA) (Fig. 2). It is measured 7.6 × 6 × 7 mm (TR x AP x CC) with focal thickening and enhancement of the left oculomotor nerve. No subarachnoid hemorrhage identified in CT or MRI images.

Our patient was diagnosed in our institution then she was referred to a tertiary referral hospital for further appropriate management. As most aneurysms can be treated surgically or with endovascular techniques.

## Discussion

The oculomotor nerve palsy is a rare neurological deficit; however, it is associated with numerous underlying pathologies. including stroke, neoplasms, trauma, post-surgical inflammation, and microvascular damage from chronic disease, considering that microvascular damage being the most common [11]. rarer presentations are uncal herniation and, least commonly (5%), cavernous sinus lesions (including tumor, vascular pathologies) [3].

Although Posterior communicating artery aneurysm is well-known to cause third cranial nerve palsy it still relatively rare in presentation, one of the studies shows that between 1985 and 2001, only 16 patients presented with third nerve palsy among 171 patients had an unruptured posterior communicating artery aneurysm smaller than 1 cm in diameter [12].

Our case is different and it is rarer, the patient has an aneurysm located at the cavernous sinus and compressing the left oculomotor nerve. Literature review shows that cavernous carotid aneurysms account for 2%-9% of all intracranial aneurysms and 15% of those originated in the internal carotid artery [1]. Lesions within the cavernous sinus can cause cavernous sinus syndrome and it can cause multiple nerve palsies due to the anatomical constituents of the oculomotor, trochlear, ophthalmic, and maxillary divisions of trigeminal and abducens.

Evaluation of the cranial nerve is important especially with oculomotor nerve palsy as imaging is often the most reliable diagnostic modality in these conditions, our patient presented with images verified aneurysm with symptoms congruent with this finding, which make this case unique. Early recognition and evaluation of the third cranial nerve palsy is important in order to rule out potential aneurysms.

Utilization of conventional Magnetic resonance imaging (MRI) imaging sequences, it could be difficult to assess the cisternal segments of the cranial nerves, which are small in diameter and situated in closeness to numerous other anatomic structures.

Steady-state free precession sequences usually referred to by their trade names or abbreviations like fast imaging employing steady-state acquisition which called fast imaging employing steady-state acquisition (FIESTA) (in GE MRI machine), and constructive interference steady state which called constructive interference steady state (CISS) (in siemens MRI machine). depict these nerve segments in greater detail and can give significant information about the relationship of the nerves to numerous pathologies. These sequences are high-resolution T2 weighted imaging with 3D multiplanar reconstruction. To take full advantage of this information, radiologists should be familiar with the normal nerve anatomy and relevant anatomic landmarks.

This case illustrates the complexity of the cavernous sinus and demonstration of the critical role of neurovascular imaging techniques as it is considered to be the diagnostic gold standard. Radiological evaluation of third cranial nerve palsies remains a difficult task and should be done by a sub-specialized experienced radiologist.

## Conclusion

Oculomotor cranial nerve palsy is a rare neurological deficit. Posterior communicating artery aneurysm is well-known to cause third cranial nerve palsy. However, there are few articles published regarding aneurysms arising from the cavernous portion of the internal carotid artery and causes oculomotor cranial nerve palsy

Early recognition and evaluation of third cranial nerve palsy is important in order to prevent complications and improve postoperative recovery. This case demonstrates the critical role of neurovascular imaging techniques as it considered the diagnostic gold standard.

## Patient consent

Verbal and written informed consent were obtained from the patient for their anonymized information to be published in this article. This report does not contain any personal information that could lead to the identification of patient.

## Authorship

All authors attest that they meet the current ICMJE criteria for authorship.
